# The Biosynthesis of Bacterial Cellulose Composites Accompanied by Spray Feeding of Biomasses

**DOI:** 10.3390/polym16172541

**Published:** 2024-09-08

**Authors:** Jiali Xu, Xiaodi Liu, Qiang Zhang

**Affiliations:** Shanghai Key Laboratory of Regulatory Biology, School of Life Sciences, East China Normal University, Shanghai 200241, China; xjl2549@163.com (J.X.); xdliu@stu.ecnu.edu.cn (X.L.)

**Keywords:** bacterial cellulose, biomass nanofiber, composite materials, high yield, functionalization

## Abstract

Bacterial cellulose (BC) is a broadly utilized natural nanofiber produced by microbial fermentation, but its high-cost and low-yield production and limited function still hinder its application. Here, we used the spraying-assisted biosynthesis method to introduce biomass nanofibers along with the nutrient media to the fermenting BC. Biomass nanofibers could be cellulose, chitosan, and others. They entangled with BC nanofibers via intermolecular interactions, including hydrogen binding and electrostatic adsorption, to form uniform BC composites. The BC composites achieved an enhanced yield of ~140 wt% compared with pure BC and displayed similar excellent mechanical properties (Young’s moduli = 0.9–1.4 MPa for wet films and =~6500 MPa for dried films). BC composites also had similar high crystallinity and thermal stability to pure BC. The functional groups of biomasses endowed BC composite additional functions such as antibacterial and dye-adsorption capabilities. Moreover, a high yield and functionalization could be realized simultaneously by feeding functional cellulose nanofibers. This method provides a facile way to produce BC composites with low cost, high yield, and multiple functions.

## 1. Introduction

Bacterial cellulose (BC) is a natural cellulose nanofiber produced by diverse bacterial species like the Gram-negative genus *Komagataeibacter* [1]. These bacteria polymerize and secrete linear chains of glucose that self-assemble into nanofibers of 50–120 nm [2]. The nanofibers in situ interweave into a three-dimensional network structure that floats at the air–media interface for food colonization and protecting the growing bacteria against environmental hazards [3]. Compared with plant cellulose, BC has a series of advantages, including high purity, high degree of polymerization, high crystallinity, high specific area, high thermal stability, great water-holding capability, excellent mechanical properties, and inherent biocompatibility [4,5]. For instance, BC has a maximum thermal degradation rate temperature of up to 355 °C and can hold ~99% water in its network [6]. The individual BC nanofiber is estimated to have a high tensile strength of ~2 GPa and a high Young’s modulus of ~138 GPa [7]. These advantages make BC a promising raw material for a wide range of applications involving food additives, wound dressing, surgical meshes, scaffolds for tissue engineering, wearable electronics, face masks, artificial leather, and so on [8,9]. BC has been commercially produced for several decades due to its diverse applications, but the issues of high production cost, yield limitation, and functional modification still hinder its utilization in broader applications [10].

To improve BC production, researchers have made continuing efforts in the filtration of preponderant bacterial strains, seeking low-cost nutrition sources and improving the fermentation parameters [11,12]. So far, a large number of BC producers such as *Gluconacetobacter*, *Azotobacter*, *Rhizobium*, *Lactobacillus*, and *Sarcina* have been isolated [13]. Among them, *Gluconacetobacter xylinus* (*G. xylinus*), which initially produces relatively large amounts of BC from a wide range of carbon and nitrogen sources, is most commonly studied [14]. Some new BC-producing strains are also isolated from rotten fruits, bean roots, etc., with high yields of BC [15]. Recently, some researchers have employed genetic engineering strategies to improve BC production [16,17]. Except for the strains, researchers have also devoted great effort to improving the carbon source, culture parameters, and fermentation methods to achieve the high-yield production of BC [18,19,20]. To date, BC can be commercially produced at a high yield of >10 g/L in 7–14 days [21].

Except for the low-cost and high-yield BC production, BC modified with different properties is also widely investigated to realize its potential applications [22,23]. In general, BC modification can be performed by either in situ modification during fermentation or the ex situ modification of the as-generated BC gels [24]. In the in situ modification, additives such as collagen, chitosan, hydroxyapatite, silica, and carbon nanotube are added in the culture media, which entangle with BC nanofibers in the network via physical interactions like hydrogen binding, hydrophobic interaction, and electrostatic absorption [25,26,27,28,29]. An automatic spray system is further developed to continuously feed additives such as graphene in BC gels, producing highly uniform BC composite gels [30,31]. The ex situ modification can be physical or chemical during which additives diffuse or absorb in the BC network during incubation [32,33]. Recently, genetic engineering methods have been employed to endow BC producers with the additional capability to produce BC nanofibers with new contents. For instance, Kenneth et al. genetically engineered *Komagataeibacter rhaeticus* to create a bacterial strain that grows self-pigmenting BC [21]. Charlie et al. developed a method to fabricate functional BC living materials via a genetically engineered co-culture of *Komagataeibacter rhaeticus* bacteria and *Saccharomyces cerevisiae* yeast [3]. However, these modification methods are either inefficient or conducted difficultly. 

Although many strategies have been developed for improving BC production and functionalization, these methods mostly suffer from low efficiency, high cost, complicated processes, and/or yield limits. Moreover, yield enhancement and functionalization are generally realized via two individual approaches. In this study, we developed a spraying-assisted biosynthesis method that produced BC composites with both yield enhancement and functionalization. The method involved the simultaneous growth of BC via static fermentation and the co-deposition of functional biomass-based nanofibers (BMNFs) through electrically controlled and time-interval spaying. The as-generated BC/BMNF composites accomplished the goals of enhanced yields and modifications at once without consuming additional nutrients. Moreover, the mechanical properties and thermal stability of BC/BMNF composites had no distinguishing difference from pure BC. The method provides a facile and robust way to produce BC/BMCNF composites with relatively high yield and additional functionality.

## 2. Materials and Methods

### 2.1. Materials and Reagents

Wood pulps were provided by Shandong Senxin Environmental Protection Technology Co., Ltd. (Taian, China). Cellulose enzyme was provided by XiaSheng Biotechnology Development Co., Ltd. (Beijing, China). Sodium hydroxide (NaOH, ≥98%), hydrochloric acid, ethanol (≥99.8%), methylene blue (MB, 95%), and acetic acid (99.5%) were obtained from Shanghai Titan Technology Co., Ltd. (Shanghai, China). Chloroacetic acid was purchased from Shanghai Aladdin Biochemical Technology Co., Ltd. (Shanghai, China). 5-([4,6-dichlorotriazine-2-yl] amino) fluorescein (DTAF) was bought from Sigma-Aldrich (St. Louis, MO, USA). Chitosan nanofibers (CSNFs) were obtained from Shanghai Mujing Material Co., Ltd. (Shanghai, China). Chitin was provided by Shanghai Maclean Biochemical Technology Co., Ltd. (Shanghai, China).

### 2.2. Preparation of Cellulose Nanofibers (CNFs)

CNFs were synthesized according to the previously reported method [34]. In a typical assay, 10 g bleached wood pulp powders were added to a 1000 mL cellulase aqueous solution (10 mg/mL, pH = 5, adjusted using 5 wt% NaOH), and the mix was incubated at 70 °C for 24 h. The product was washed 6 times via centrifugation (7800 rpm, 10 min) and then suspended in the deionized (DI) water (1 wt%). The wood pulp suspension was further treated with an ultrasonic homogenizer (20 min, 1200 W, Ningbo Scientz Biotechnology Co., Ltd., Ningbo, China) and a high-pressure homogenizer 3 times (100 mPa, AH-PILOT 2018, ATS Engineering Limited, Suzhou, China) to obtain CNFs.

### 2.3. Preparation of Carboxymethyl Cellulose Nanofibers (CMCNFs)

CMCNFs were prepared via a typical method [35]. Briefly, 3 g of bleached wood pulp powder was suspended in 200 mL ethanol under magnetic stirring. Then, 5 mL of NaOH (50 wt%) aqueous solution was added to alkalize the wood pulp for 1 h at room temperature. After that, 10 mL of chloroacetic acid in ethanol solution (50 wt%) was added to the reaction system. After 4 h, 3 mL of acetic acid was added to terminate the reaction. The product was washed 5 times via centrifugation (7800 rpm, 10 min) using 60 *v*/*v*% ethanol in DI water and then suspended in DI water (1 wt%). Finally, the product was treated using an ultrasonic crusher (20 min, 1200 W) and a high-pressure homogenizer 3 times (100 MPa) to obtain CMCNFs.

### 2.4. Preparation of Chitin Nanofibers (CHNFs)

CHNFs were synthesized according to a reported method [36]. In a typical assay, 8 g of chitin was added into 400 mL hydrochloric acid (3 mol/L) solution. Subsequently, the above solution was heated at 100 °C for 4 h under vigorous stirring. Then, the sample was washed with DI water via centrifugation (7800 rpm, 10 min) until the pH was neutral. Finally, the sample was treated by an ultrasonic crusher (20 min, 1200 W) and a high-pressure homogenizer 3 times (100 MPa) to obtain CHNFs.

### 2.5. Preparation of CMCNF-Fluo

Typically, 1 g of NaOH was added to 500 mL of CMCNF suspension (1 wt%), and then 10 g of DTAF was added. The reaction was carried out at room temperature for 24 h under dark conditions. The product was purified via centrifugation (7800 rpm, 10 min).

### 2.6. Synthesis of BC/BMNF Composite Hydrogels and Films

In a typical assay, 15 g of agar (1.5 wt%) was added to 1000 mL of the Hestrin–Schramm medium. The mix was sterilized at 115 °C for 20 min and then poured into a fermentation tank and cooled at room temperature until a solid plate of 1.0 cm thickness was formed. The bacterial Acetobacter xylinum (American Type Culture Collection, Manassas, VA, USA) was inoculated onto the solid culture media in the tank, and a thin hydrogel was formed on the surface of the solid medium after 12 h incubation. After that, the culture medium containing BMNFs was sprayed in the tank at a frequency of 2 s every 2 h. 24 h later, the BC/BMNF composite hydrogel was obtained. The hydrogel was purified with a 2 wt% NaOH solution and then rinsed with DI water until the pH value became 7.0. The purified BC/BMNF composite hydrogels were dried at 60 °C to obtain the dried films.

### 2.7. Yield Measurement

The yield of BC/BMNF composite hydrogels is defined as the amount of solid produced by consuming a liter culture medium during fermentation. The BC/BMNF composite hydrogels were purified and dried into films, and then the films were weighed. The yield was calculated by the following formula:

$$Yield=M_{BC/BMNF}/V$$

where M_BC/BMNF_ is the weight of BC/BMNF composite film, and V is the volume of medium consumed during fermentation.

### 2.8. The Measurement of Apparent Utilization

The BC and BC/BMNF composite hydrogels were obtained by spraying the normal culture media and the one containing BMNFs. The apparent utilization was calculated by the following formula:

$$Apparentutilization=\frac{M_{BC/BMNF}-M_{BC}}{M_{BC}}\times 100\%$$

where M_BC/BMNF_ is the weight of BC/BMNF composite film, and M_BC_ is the weight of BC film.

### 2.9. The Measurement of Solid Content

Solid content refers to the proportion of biopolymers in the hydrogels. After washing with NaOH, the hydrogels were wiped with a paper towel to remove surface water, and their weight was measured. After that, the hydrogels were dried at 60 °C to constant weight, and the weight was recorded. The solid content was calculated by the following equation:

$$Solidcontent=\frac{M_{f}}{M_{h}}\times 100\%$$

where M_h_ is the weight of the hydrogel, and M_f_ is the weight of the corresponding dried film.

### 2.10. Characterization

The scanning electron microscopy (SEM) images were captured with a scanning electron microscope (S-4800, Hitachi, Tokyo, Japan) at an accelerating voltage of 3 kV. The Fourier transform infrared (FTIR) spectra were recorded using a Fourier transform infrared spectroscope (Nicolet iS50, Thermo Fisher Scientific, Waltham, MA, USA) within a wavenumber ranging from 550 to 4000 cm^−1^. The thermal stability of materials was measured using a thermal gravimetric analyzer (TGA4000, PerkinElmer, Waltham, MA, USA).

### 2.11. Crystallinity Index (CrI)

The X-ray diffraction (XRD) patterns were recorded with an X-ray diffractometer (Smartlab SE, Rigaku, Japan) within a 2θ ranging from 8 to 40°. The CrI of cellulose was calculated according to the peak height in the XRD spectra; the equation is as follows [37]:

$$\mathrm{CrI}=\frac{I200-Iam}{I200}\times 100\%$$

where *I*_200_ is the maximum intensity corresponding to the 200 plane, and *I_am_* is the intensity of diffraction at 2θ ≈ 18° equivalent to amorphous cellulose.

The CrI of chitosan was calculated according to the following equation [38]:

$$\mathrm{CrI}=\frac{I110-Iam}{I110}\times 100\%$$

where *I*_110_ is the maximum intensity corresponding to the 110 plane, and *I_am_* is the intensity of diffraction at 2θ ≈ 16° caused by amorphous chitosan.

### 2.12. Mechanical Property Measurement

The mechanical properties were measured with a universal testing machine (Hengyi Precision Instrument Co., Ltd., Shanghai, China) in tensile mode. Both hydrogels and films were cut into long strips. Young’s modulus, tensile strength, and percentage elongation at break were calculated from the stress–strain data. Five specimens were measured to obtain the average values.

### 2.13. Cell Culture

NIH/3T3 cells (CLS GmbH, Eppelheim, Germany) were cultured in the Dulbecco’s modified Eagle medium (DMEM, high glucose, Gibco, Waltham, MA, USA) supplemented with 10% fetal bovine serum (Gibco, Waltham, MA, USA) and 1% penicillin–streptomycin (Sigma-Aldrich, St. Louis, MO, USA) at 37 °C under 5% CO_2_.

### 2.14. Cell Viability Assay

Cell viability was determined by 3-(4,5-dimethylthiazol-2-yl)-2,5-diphenyltetrazolium bromide (MTT) assay. The leachates of BC/CNF, BC/CMCNF, and BC/CSNF composite hydrogel were prepared into different concentrations with the DEME medium. NIH3T3 cells were incubated with BC/CNF, BC/CMCNF, and BC/CSNF leachates for 24 h at 37 °C, and then the cell viabilities were determined by MTT assay. 

### 2.15. Acridine Orange/Ethidium Bromide (AO/EB) Staining Assay

NIH3T3 cells were placed in a 24-well plate and cultured for 24 h. The leachates of BC/CNF, BC/CMCNF, and BC/CSNF composite hydrogels were added to replace the old culture media. After 24 h incubation, the medium was removed, and 500 μL AO/EB dye (50 μg/mL) was added to each well. The cells were stained at 37 °C for 10 min. Finally, the dye was discarded, and the wells were observed under a fluorescence microscope.

### 2.16. Antibacterial Assessment

The bacterial strains used for the experiment were Escherichia coli (*E. coli*, ATCC) and Staphylococcus aureus (*S. aureus*, ATCC). The BC/CSNF hydrogel was prepared into 1% suspension and sterilized at a high temperature (121 °C, 20 min). After the resuscitation, the bacteria strains were co-cultured with the BC/CSNF suspension for 16 h at 37 °C. The samples in each group were diluted 10^9^ times in a 96-well plate. Then, 10 μL of each gradient sample was evenly diluted and placed on LB agar plates. After 18 h incubation, the colonies on the agar were counted visually and as CFU per sample. 

### 2.17. Dye-Adsorption Capability

In a typical assay, 2.5 g/L of MB standard stock solution was prepared and stored at 4 °C in the dark. Then, 100 mg of dried BC or BC/CMCNF film was added in 50 mL centrifuge tubes, and 10 mL of MB solution (12.5 mg/L) was added. The tubes were shaken at 30 °C and 100 r/min for 60 min. Finally, the solution was filtered and measured at 665 nm using an ultraviolet spectrophotometer (UV-specoRD210 plus, Analytik, Jena, German). The concentration of MB was calculated according to the calibration curve.

## 3. Results and Discussion

**Figure 1:**
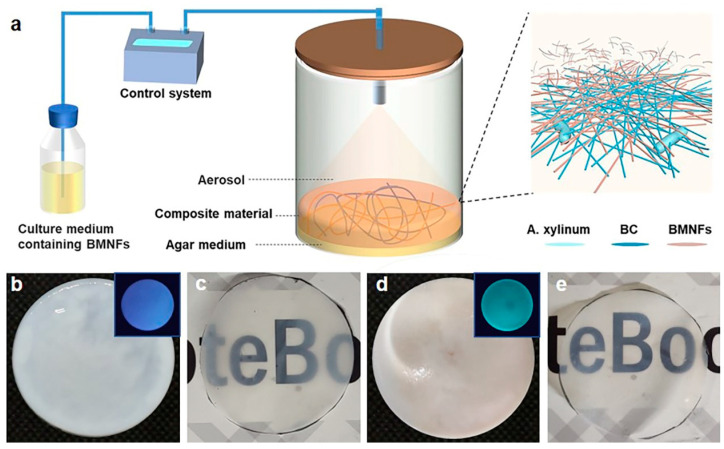
Synthesis of BC/BMNF composite hydrogels: (**a**) schematic illustration of fermentation of BC/BMNF composite hydrogels; BMNFs were suspended in the culture media and supplied to BC fermentation; (**b**,**c**) the pure BC hydrogel (**b**) and film (**c**); (**d**,**e**) BC/CMCNF hydrogel (**d**) and film (**e**). Insets in (**b**,**d**) are fluorescent images of the corresponding hydrogels illuminated with ultraviolet light. CMCNFs were stained by green fluorescence.

To achieve our goal, a homemade static fermentation system was used (Figure 1a). A transparent fermentation cylinder was employed for the static culture of BC, and a mix of the basic nutrient culture media and additives was intermittently spayed into the fermentation cylinder from the top. The additives in our study were BMNFs including CNFs, CMCNFs, CSNFs, and CHNFs (Figures S1–S4). CNFs were cellulose nanofibers without chemical modification, while CMCNFs were cellulose nanofibers modified with carboxymethyl groups. CSNFs were chitosan nanofibers that were made of chitosan with 95% deacetylation (Figure S1). Since BC nanofibers were produced at the interface of air–medium, the BMNFs floated down along with the culture media and entangled with the in situ-generated BC nanofibers via interactions mainly involving hydrogen binding, hydrophobic interactions, and electrostatic adsorption. The optimized spraying time was 2 s, and the interval time was 2 h (Table S1), under which condition an intact BC composite hydrogel without layered structure was obtained. As a result, the added BMNFs were perfectly integrated into the BC network. The pure BC hydrogel was translucent white, and the BC/CMCNF hydrogel was opaque white (Figure 1b,d). CMCNFs were modified using green fluorescence to detect their presence in the composite hydrogel. The inset images in Figure 1b,d show that the BC/CMCNF hydrogel emitted green fluorescence upon ultraviolet light illumination, while pure BC hydrogel did not. After drying, both BC and BC/CMCNF films presented similar transparencies (Figure 1c,e).

**Figure 2:**
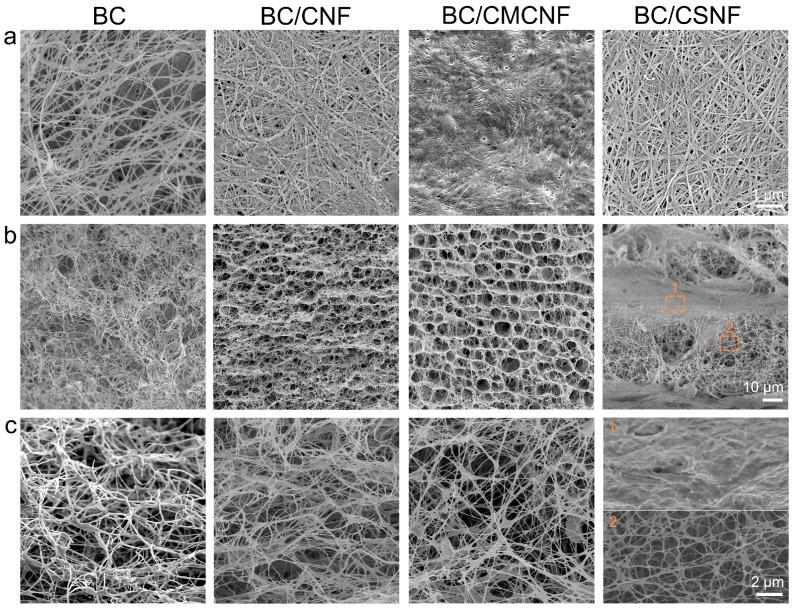
SEM images of BC/BMNF hydrogels: (**a**) the surface and (**b**,**c**) cross-section images of BC, BC/CNF, BC/CMCNF, and BC/CSNF hydrogels.

The microstructure of BC and BC/BMNF hydrogels was investigated using a scanning electron microscope. The densities of nanofibers in the surface images of hydrogels were quite different (Figure 2a). The BC hydrogel had the lowest fiber density, and the BC/CMCNF hydrogel had the highest density. Moreover, the arrangement orientations of the nanofibers in these hydrogels were also distinguished. The fibers in the BC hydrogel were randomly entangled with others, leading to gaps of varied sizes. In comparison, the arrangement of nanofibers in the other three composite hydrogels looked more orderly than that in the BC hydrogel. In particular, in the BC/CSNF hydrogel, the fibers were straighter and interweaved orderly. The cross-section images show that the microstructure of BC/BMNF hydrogel was quite different from that of BC (Figure 2 and Figure S4). The BC hydrogel was highly porous and had a homogenous microstructure. In comparison, BC/CNF and BC/CMCNF hydrogels possessed a layer-by-layer structure and had uniform micropores among the layers. The addition of CNFs and CMCNFs instantly increased the amount of biomass at the fermentation interface, leading to the formation of layer structure. The cross-section of BC/CSNF and BC/CHNF hydrogels exhibited a thicker layer compared with the one in BC/CNF and BC/CMCNF hydrogels (Figure 2 and Figure S4). BC hydrogels were generally fermented in a slightly acidic media [39]. CSNFs and CHNFs had positive surface charge in the slightly acidic fermentation broth [40], which promoted the electrostatic interaction with BC and thus led to the formation of thicker layer composites in their structure. These data suggest that the added BMNFs had a significant influence on the microstructure of BC composite hydrogels. 

**Figure 3:**
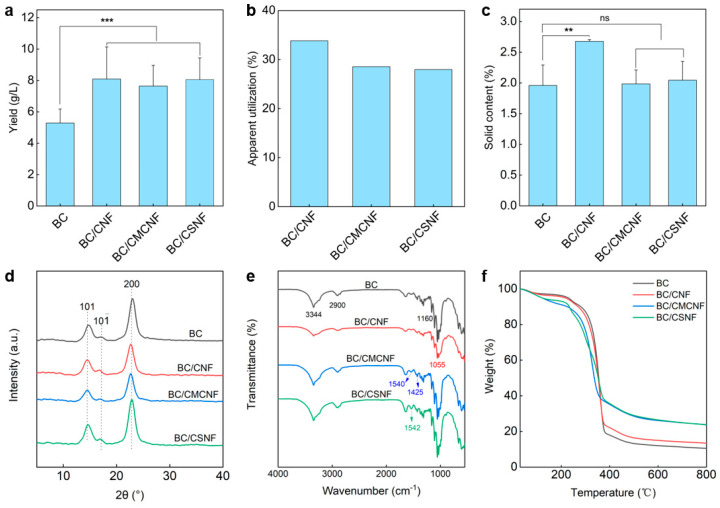
Characterization of BC/BMNF hydrogels: (**a**) yields of BC/BMNF hydrogels; (**b**) the apparent utilization of BMNFs during the fermentation; (**c**) the solid content of BC/BMNF hydrogels; (**d**,**e**) XRD and FTIR spectra of BC/BMNF hydrogels; (**f**) the thermogravimetric analysis of BC/BMNF hydrogels. n.s. means no significance, ** means *p* < 0.01, and *** means *p* < 0.001 analyzed by Student’s *t*-test.

Since the additional nanofibers were introduced into the BC network, the apparent yield of BC/BMNF hydrogels should be increased. To address these issue, BC and BC/BMNF hydrogels were cultured at the same condition except for adding BMNFs and were then assessed for their dry weight. As shown in Figure 3a, the BC hydrogel had a yield of 5.3 ± 0.9 g/L, while the BC/BMNF hydrogels had yields of more than 7.5 ± 1.6 g/L, which was 1.4 times the yield of BC. For instance, the BC/CNF hydrogels had a highest yield of 8.1 ± 2.0 g/L. These data suggest that the composite hydrogels had a higher yield regardless of whether the fibers were BC or the added BMNFs. Moreover, the apparent utilization of BMNFs was calculated. In this case, we presumed that BC nanofibers in BC/BMNF hydrogels had an equal mass despite the added BMNFs that might influence BC fermentation. The apparent utilization rate of BMNFs was measured to be 28–34% (Figure 3b). The low apparent utilization rates of BMNFs should be attributed to the altered fermentation environment caused by adding BMNFs and alkali washing caused the loss of BMNFs in the as-obtained BC composite hydrogel. The solid contents of BC/BMNF hydrogels were also determined. The BC/CNF hydrogel possessed a relatively high solid content of 2.7 ± 0.03 wt%, while BC, BC/CMCNF, and BC/CSNF hydrogels had a solid content of ~2 wt% (Figure 3c). CNFs had the same chemical structure as BC, and thus they were more compatible with BC than CMCNFs or CSNFs. The XRD patterns reveal that the three BC/BMNF hydrogels had the same characteristic peaks at 14.6° and 22.94°, corresponding to the (110) and (200) planes of the cellulose crystal structure, respectively [41]. This may explain why BC/CNF and BC/CMCNF hydrogels had the same XRD peaks as BC hydrogel, as they were cellulose (Figure 3d). However, the BC/CSNF hydrogel also had the same characteristic peaks as the BC hydrogel, which is probably due to a small number of CSNFs were entangled in the BC network (Figure 3d). The BC hydrogel had a high CrI of 96.2%, while the BC/BMNF hydrogel possessed a smaller CrI of around 95.0% as the added BMNFs had relatively low CrI (Figure 3d and Figure S2). The FTIR spectra reveal that the BC/BMNF hydrogel had the typical absorbance bands for these added BMNFs (Figure 3e). For BC and BC/CNF, the absorbance bands at 3344 cm^−1^ and 2990 cm^−1^ were assigned to O-H and C-H stretching vibrations, respectively [42]. The BC/CNF hydrogel had the typical absorbance band at 1055 cm^−1^, which was attributed to the bending of the C-O-H bond [43]. The characteristic absorbance bands of the BC/CMNF hydrogel appeared at 1540 and 1425 cm^−1^ assigned to the asymmetric and symmetric stretching vibrations of -COO^−^, respectively, corresponding to the -CH_2_COO^−^ group in CMCNFs [44]. The BC/CSNF hydrogel had a weak absorbance band at 1595 cm^−1^, corresponding to the fingerprint peak absorption of N-H bending in chitosan [45], indicating the successful introduction of CSNFs in BC/CSNF. The thermogravimetric analysis and derivative thermogravimetric curves reveal that the thermal decomposition of the BC and BC/BMNF films was divided into three stages (Figure 3f and Figure S5). The initial ~7 wt% mass losses in the temperature range of 25–252 °C were the evaporation of adsorbed water (Figure 3f). The second stage of mass loss in the temperature range of 252–387 °C was due to the decomposition of cellulose (Figure 3f). The maximum decomposition rate temperature was 361 °C for the BC film (Figure S5). The temperatures for BC/CNF and BC/CSNF films were 344 and 356 °C, respectively (Figure S5), which were similar to that of the BC film. However, the temperature for BC/CMCNF had two maximum decomposition rate temperatures at 241 and 345 °C (Figure S5). The decreased temperature may be because CMCNFs were not thermally stable. In the third stage, the mass losses were attributed to the carbonization of the cellulose skeleton.

**Figure 4:**
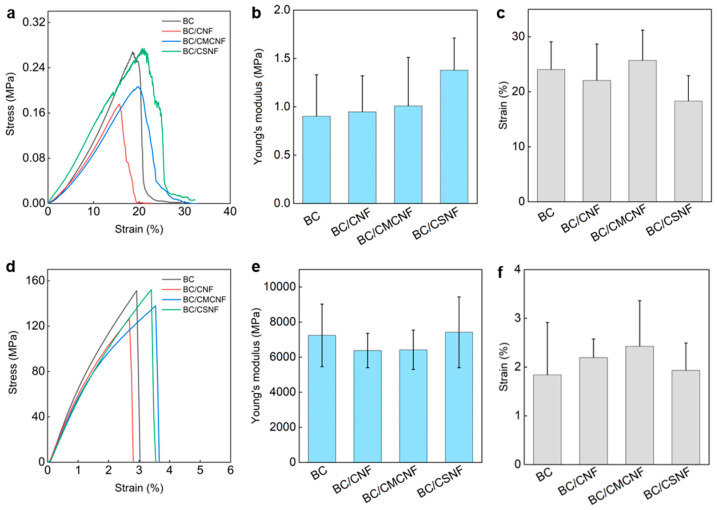
Mechanical properties of BC/BMNF hydrogels and films: (**a**) the tensile stress–strain curves of BC/BMNF hydrogels and (**b**,**c**) their corresponding Young’s moduli and strains; (**d**) the tensile stress–strain curves of BC/BMNF films and (**e**,**f**) their corresponding Young’s moduli and strains.

The mechanical properties of BC/BMNF hydrogels and films were investigated. The BC hydrogel had a tensile stress of 0.27 MPa, and the BC/BMNF hydrogels had a similar tensile stress to the BC one (Figure 4a). The Young’s moduli of BC/BMNF hydrogels also had no obvious difference from that of the BC hydrogels, which ranged from 0.95 ± 0.37 to 1.38 ± 0.33 MPa (Figure 4b). The BC/BMNF hydrogels also had similar strains to the BC ones, which were around 24.04 ± 5.04% (Figure 4c). Both the concentrations of CNFs and CMCNFs in the spaying culture media varied, which did not influence the mechanical properties of the as-obtained composite hydrogel (Figures S6 and S7). As the BC and BC/BMNF hydrogels had similar solid contents of around 2 wt%, and BMNFs were well integrated into the BC network, mainly via hydrogen binding, it was reasonable that BC/BMNF hydrogels had similar mechanical properties with BC hydrogels. After drying, the tensile stresses of the films were also measured. The BC and BC/BMNF films had similar tensile stresses, which were larger than 120 MPa (Figure 4d). Their Young’s moduli were around 6500 MPa, and their strains were around 2% (Figure 4e,f). The concentrations of CNFs and CMCNFs in the spaying culture media also did not influence the mechanical properties of the as-obtained composite films (Figures S6 and S7). Therefore, the added BMNFs also did not influence the mechanical properties of the composite films as the inter- and intra-molecular interactions of the fibers in the pure BC and BC/BMNF composite were similar. 

**Figure 5:**
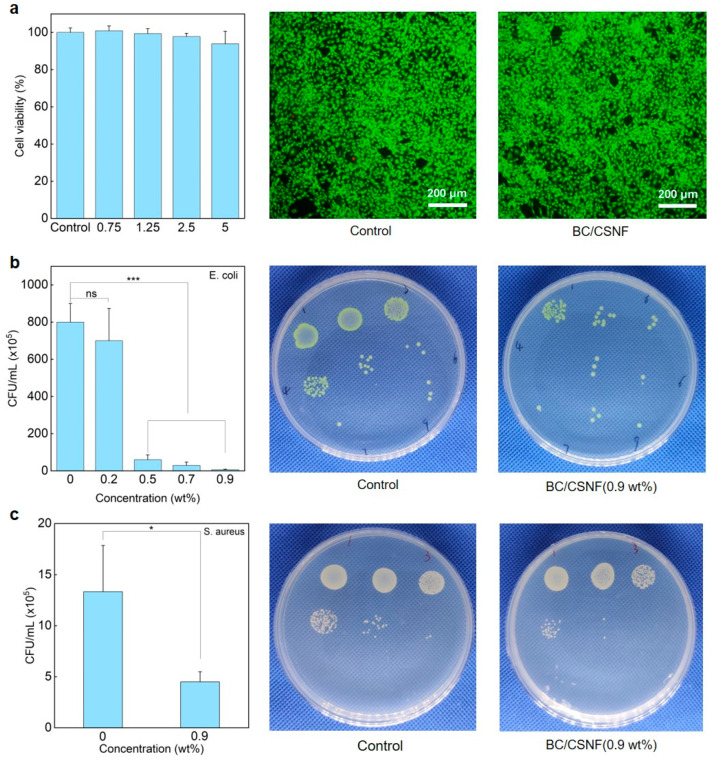
Cytotoxicity and antibacterial ability of BC/CSNF hydrogels: (**a**) cytotoxicity of BC/CSNF hydrogels; NIH/3T3 cells were incubated with the leachate of BC/CSNF hydrogels for 24 h; (**b**,**c**) the bacterial survival of *E. coli* (**b**) and *S. aureus* (**c**) after being treated with different concentrations of BC/CSNF for 24 h. n.s. means no significance, * means *p* < 0.05, and *** means *p* < 0.001 analyzed by Student’s *t*-test.

The biocompatibility and functions of the BC/BMNF hydrogels were evaluated. The MTT and AO/EB staining assays reveal that the BC/CNF, BC/CMCNF, and BC/CSNF hydrogels had minimal cytotoxicity (Figure 5a and Figure S8). Since chitosan possesses natural antibacterial properties [46], the BC/CSNF hydrogels were assessed for their antibacterial capability. The leachate of the BC/CSNF hydrogels was used to culture cells. Furthermore, the antibacterial capability of the BC/CSNF hydrogels was evaluated. When the concentration of the BC/CSNF solution was 0.2 wt%, the inhibition ratio of *E. coli* was 37% (Figure 5b). When the concentration of BC/CSNF was 0.9 wt%, the inhibition ratio of *E. coli* was nearly 100% (Figure 5b). Moreover, the inhibition ratio of *S. aureus* was determined to be 75% when the concentration of BC/CSNF was 0.9 wt% (Figure 5c). This result suggests that the BC/CSNF hydrogels possess excellent antibacterial capability. Furthermore, the dye-absorption capability of the BC and BC/CMCNF hydrogels was assessed. MB is widely used in dyeing industries, but it is toxic and non-biodegradable [47]. The results show that both BC and BC/CMCNF hydrogels could adsorb MB (Figure S9). BC had a large number of hydroxyl groups, allowing it to adsorb MB [48]. The removal rate of MB by the BC/CMCNF hydrogel was 1.5 times higher than that of BC as CMCNFs provided plentiful carboxyl groups to interact with MB (Figure S9) [49].

## 4. Conclusions

In summary, we developed a biosynthesis method to produce BC/BMNF composite hydrogels/films with low cost, high yield, and varied functions. The method involved a static fermentation to produce BC, while BMNFs like CNFs, CMCNFs, and CSNFs were supplied along with the culture media via an intermittent spaying way. BMNFs were directly incorporated into the BC network and entangled with BC nanofibers via various physical interactions. As a result, the as-obtained BC composites provided a relatively higher yield than the pure BC and had similar mechanical properties to the pure BC. Moreover, the BC hydrogels/films could be easily functionalized by using BMNFs with natural functional groups such as CSNFs to illustrate the antibacterial ability or BMNFs pre-functionalized such as CMCNFs to endow the composites with dye-adsorption capability. This method provides a new way to produce BC composites with high yield, low cost, and multiple functions.

## Data Availability

Data are included in the article; please contact the corresponding author for further information.

## Supplementary Materials

The following supporting information can be downloaded at: https://www.mdpi.com/article/10.3390/polym16172541/s1, Table S1: effect of spraying and interval times on the synthesis of BC/CNF hydrogels; Figure S1: SEM images of (a) CNFs, (b) CMCNFs, and (c) CSNFs; Figure S2: XRD spectra of CNFs, CMCNFs, and CSNFs; Figure S3: FTIR spectra of CNFs, CMCNFs, and CSNFs; Figure S4: synthesis of BC/CHNF hydrogel; Figure S5: the derivative thermogravimetry curves of BC/BMNF composite hydrogels; Figure S6: mechanical properties of BC/CNF hydrogels; Figure S7: mechanical properties of BC/CMCNF hydrogels; Figure S8: cytotoxicity of BC/CNF and BC/CMCNF hydrogels evaluated via (a) MTT and (b) AO/EB staining assays; Figure S9: dye-adsorption capability.

## References

[1] Saleh A., El-Gendi H., Soliman N., El-Zawawy W., Abdel-Fattah Y. (2022). Bioprocess development for bacterial cellulose biosynthesis by novel *Lactiplantibacillus plantarum* isolate along with characterization and antimicrobial assessment of fabricated membrane. Sci. Rep..

[2] Almihyawi R., Musazade E., Alhussany N., Zhang S., Chen H. (2024). Production and characterization of bacterial cellulose by *Rhizobium* sp. isolated from bean root. Sci. Rep..

[3] Gilbert C., Tang T., Ott W., Dorr B., Shaw W., Sun G., Lu T., Ellis T. (2021). Living materials with programmable functionalities grown from engineered microbial co-cultures. Nat. Mater..

[4] Girard V., Chaussé J., Vermette P. (2024). Bacterial cellulose: A comprehensive review. J. Appl. Polym. Sci..

[5] Liu X., Cao L., Wang S., Huang L., Zhang Y., Tian M., Li X., Zhang J. (2023). Isolation and characterization of bacterial cellulose produced from soybean whey and soybean hydrolyzate. Sci. Rep..

[6] Volova T., Prudnikova S., Kiselev E., Nemtsev I., Vasiliev A., Kuzmin A., Shishatskaya E. (2022). Bacterial cellulose (BC) and BC composites: Production and properties. Nanomaterials.

[7] Potočnik V., Gorgieva S., Trček J. (2023). From nature to lab: Sustainable bacterial cellulose production and modification with synthetic biology. Polymers.

[8] Pandey A., Singh M., Singh A. (2024). Bacterial cellulose: A smart biomaterial for biomedical applications. J. Mater. Res..

[9] Gregory D., Tripathi L., Fricker A., Asare E., Orlando I., Raghavendran V., Roy I. (2021). Bacterial cellulose: A smart biomaterial with diverse applications. Mat. Sci. Eng. R..

[10] Wang J., Tavakoli J., Tang Y. (2019). Bacterial cellulose production, properties and applications with different culture methods—A review. Carbohydr. Polym..

[11] El-Gendi H., Taha T., Ray J., Saleh A. (2022). Recent advances in bacterial cellulose: A low-cost effective production media, optimization strategies and applications. Cellulose.

[12] Abol-Fotouh D., Hassan M., Shokry H., Roig A., Azab M., Kashyout A. (2020). Bacterial nanocellulose from agro-industrial wastes: Low-cost and enhanced production by *Komagataeibacter saccharivorans* MD1. Sci. Rep..

[13] Piwowarek K., Lipińska E., Kieliszek M. (2023). Reprocessing of side-streams towards obtaining valuable bacterial metabolites. Appl. Microbiol. Biotechnol..

[14] Yang L., Zhu X., Chen Y., Wang J. (2024). Enhanced bacterial cellulose production in *Gluconacetobacter xylinus* by overexpression of two genes (bscC and bcsD) and a modified static culture. Int. J. Biol. Macromol..

[15] Urbina L., Corcuera M., Gabilondo N., Eceiza A., Retegi A. (2021). A review of bacterial cellulose: Sustainable production from agricultural waste and applications in various fields. Cellulose.

[16] Lu T., Gao H., Liao B., Wu J., Zhang W., Huang J., Liu M., Huang J., Chang Z., Jin M. (2020). Characterization and optimization of production of bacterial cellulose from strain CGMCC 17276 based on whole-genome analysis. Carbohy. Polym..

[17] Florea M., Hagemann H., Santosa G., Abbott J., Micklem C., Spencer-Milnes X., de Arroyo Garcia L., Paschou D., Lazenbatt C., Kong D. (2016). Engineering control of bacterial cellulose production using a genetic toolkit and a new cellulose-producing strain. Proc. Natl. Acad. Sci. USA.

[18] Zahan K., Pa’e N., Muhamad I. (2015). Monitoring the effect of pH on bacterial cellulose production and Acetobacter xylinum 0416 growth in a rotary discs reactor. Arab. J. Sci. Eng..

[19] Jiang W., Jiang Z., Zhu M., Fan X. (2022). Oriented bacterial cellulose for achieving high carbon yield through pre-stretching. Cellulose.

[20] Zeng X., Small D., Wan W. (2011). Statistical optimization of culture conditions for bacterial cellulose production by *Acetobacter xylinum* BPR 2001 from maple syrup. Carbohy. Polym..

[21] Walker K., Li I., Keane J., Goosens V., Song W., Lee K., Ellis T. (2024). Self-pigmenting textiles grown from cellulose-producing bacteria with engineered tyrosinase expression. Nat. Biotechnol..

[22] Vadanan S., Basu A., Lim S. (2022). Bacterial cellulose production, functionalization, and development of hybrid materials using synthetic biology. Polym. J..

[23] Blanco P.F., Santoso S., Chou C., Verma V., Wang H., Ismadji S., Cheng K. (2020). Current progress on the production, modification, and applications of bacterial cellulose. Crit. Rev. Biotechnol..

[24] Gorgieva S., Trček J. (2019). Bacterial cellulose: Production, modification and perspectives in biomedical applications. Nanomaterials.

[25] Dhar P., Etula J., Bankar S. (2019). In situ bioprocessing of bacterial cellulose with graphene: Percolation network formation, kinetic analysis with physicochemical and structural properties assessment. ACS Appl. Bio. Mater..

[26] Gorgieva S. (2020). Bacterial cellulose as a versatile platform for research and development of biomedical materials. Processes.

[27] Choi S., Rao K., Zo S., Shin E., Han S. (2022). Bacterial cellulose and its applications. Polymers.

[28] Illa M., Peddapapannagari K., Raghavan S., Khandelwal M., Sharma S.C. (2021). In situ tunability of bacteria derived hierarchical nanocellulose: Current status and opportunities. Cellulose.

[29] Li D., Qu X., Newton S., Klebba P., Mao C. (2012). Morphology-controlled synthesis of silica nanotubes through pH-and sequence-responsive morphological change of bacterial flagellar biotemplates. J. Mater. Chem..

[30] Luo H., Dong J., Yao F., Yang Z., Li W., Wang J., Xu X., Hu J., Wan Y. (2018). Layer-by-layer assembled bacterial cellulose/graphene oxide hydrogels with extremely enhanced mechanical properties. Nano-Micro Lett..

[31] Luo H., Xiong P., Xie J., Yang Z., Huang Y., Hu J., Wan Y., Xu Y. (2018). Uniformly dispersed freestanding carbon nanofiber/graphene electrodes made by a scalable biological method for high-performance flexible supercapacitors. Adv. Funct. Mater..

[32] Cazón P., Vázquez M. (2021). Improving bacterial cellulose films by ex-situ and in-situ modifications: A review. Food Hydrocoll..

[33] Nicoara A., Stoica A., Ene D., Vasile B., Holban A., Neacsu I. (2020). In situ and ex situ designed hydroxyapatite: Bacterial cellulose materials with biomedical applications. Materials.

[34] Qi Y., Guo Y., Liza A., Yang G., Sipponen H.M., Guo J., Li H. (2023). Nanocellulose: A review on preparation routes and applications in functional materials. Cellulose.

[35] Wang L., Zhang C., Zhao W., Li W., Wang G., Zhou X., Zhang Q. (2022). Water-swellable cellulose nanofiber aerogel for control of hemorrhage from penetrating wounds. ACS Appl. Bio Mater..

[36] He Y., Zhou Y., Cai J., Feng Y., Luo B., Liu M. (2022). Facile fabrication of hydrophobic paper by HDTMS modified chitin nanocrystals coating for food packaging. Food Hydrocolloid..

[37] Salem K., Kasera N., Rahman M., Jameel H., Habibi Y., Eichhorn S., French A., Pal L., Lucia L. (2023). Comparison and assessment of methods for cellulose crystallinity determination. Chem. Soc. Rev..

[38] Harish Prashanth K., Kittur F., Tharanathan R. (2002). Solid state structure of chitosan prepared under different N-deacetylating conditions. Carbohydr. Polym..

[39] Yassine F., Bassil N., Flouty R., Chokr A., Samrani A., Boiteux G., Tahchi M. (2016). Culture medium pH influence on *Gluconacetobacter* physiology: Cellulose production rate and yield enhancement in presence of multiple carbon sources. Carbohydr. Polym..

[40] Hagar H., Jufar S., Lee J., Al-mahbashi N., Alameen M., Kwon S., Jagaba A., Rathnayake U. (2023). Chitin nanocrystals: A promising alternative to synthetic surfactants for stabilizing oil-in-water emulsions. Case Stud. Chem. Environ. Eng..

[41] Vasconcelos N., Feitosa J., da Gama F., Morais J., Andrade F., de Souza Filho M., de Freitas Rosa M. (2017). Bacterial cellulose nanocrystals produced under different hydrolysis conditions: Properties and morphological features. Carbohydr. Polym..

[42] Wu Z., Yin P., Ju H., Chen Z., Li C., Li S., Liang H., Zhu J., Yu S. (2019). Natural nanofibrous cellulose-derived solid acid catalysts. Research.

[43] Lahiri D., Nag M., Dutta B., Dey A., Sarkar T., Pati S., Edinur H., Abdul Kari Z., Mohd Noor N., Ray R. (2021). Bacterial cellulose: Production, characterization, and application as antimicrobial agent. Int. J. Mol. Sci..

[44] Qin F., Fang Z., Zhou J., Sun C., Chen K., Ding Z., Li G., Qiu X. (2019). Efficient removal of Cu^2+^ in water by carboxymethylated cellulose nanofibrils: Performance and mechanism. Biomacromolecules.

[45] Ebrahimi Tirtashi F., Moradi M., Tajik H., Forough M., Ezati P., Kuswandi B. (2019). Cellulose/chitosan pH-responsive indicator incorporated with carrot anthocyanins for intelligent food packaging. Int. J. Biol. Macromol..

[46] El-Araby A., Janati W., Ullah R., Ercisli S., Errachidi F. (2024). Chitosan, chitosan derivatives, and chitosan-based nanocomposites: Eco-friendly materials for advanced applications (a review). Front. Chem..

[47] El Jery A., Alawamleh H., Sami M., Abbas H., Sammen S., Ahsan A., Imteaz M., Shanableh A., Shafiquzzaman M., Osman H. (2024). Isotherms, kinetics and thermodynamic mechanism of methylene blue dye adsorption on synthesized activated carbon. Sci. Rep..

[48] Sivakumar R., Lee N. (2022). Adsorptive removal of organic pollutant methylene blue using polysaccharide-based composite hydrogels. Chemosphere.

[49] Chen K. (2023). Adsorption properties of carboxymethyl cellulose/carbon hydrogel for copper and methylene blue. Desalin. Water Treat..

