# Supplementary material for: The Biosynthesis of Bacterial Cellulose Composites Accompanied by Spray Feeding of Biomasses
Source: Polymers (Basel). 2024 Sep 8;16(17):2541. doi: 10.3390/polym16172541 (PMC11397784; doi:10.3390/polym16172541)
Supplement: Supplementary file 1 [file polymers-16-02541-s001.zip › polymers-3082585-supplementary.pdf]

Supporting Information

The Biosynthesis of Bacterial Cellulose Composites Accompanied by Spray Feeding of Biomasses

Jiali Xu†, Xiaodi Liu†, and Qiang Zhang*

Shanghai Key Laboratory of Regulatory Biology, School of Life Sciences, East China Normal University, Shanghai, 200241, P.R. China；xjl2549@163.com (J.X.)； xdliu@stu.ecnu.edu.cn (X.L.)

*Correspondence: qzhang@bio.ecnu.edu.cn

†The authors contributed equally to this work.

Table S1. Effect of spraying and interval times on the synthesis of BC/CNF hydrogels.

Spraying time (s)	Interval time (h)	Structure of BC/CNF hydrogels
5	3	multiple layers
4	3	multiple layers
3	3	multiple layers
2	3	single layer
1	3	single layer
2	4	single layer
2	3	single layer
2	2	single layer
2	1	multiple layers

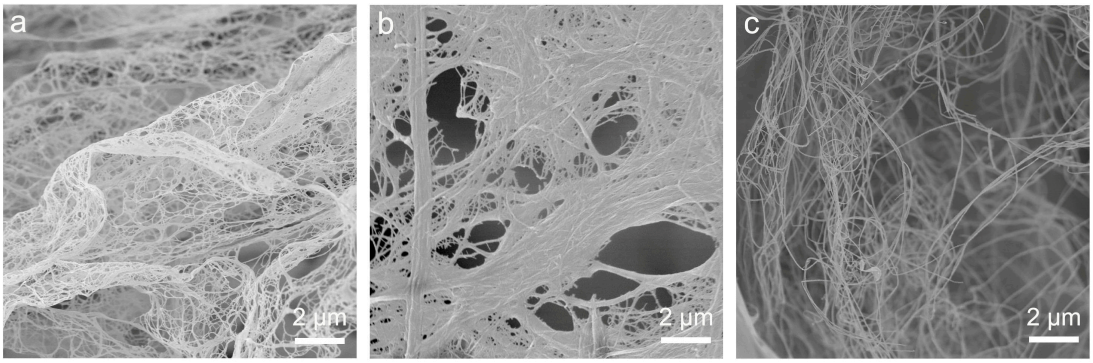

Figure S1. SEM images of (**a**) CNFs, (**b**) CMCNFs, and (**c**) CSNFs.

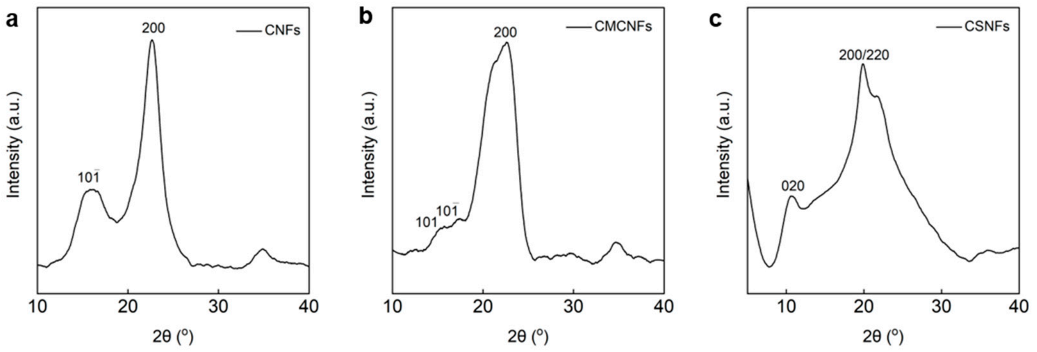

Figure S2. XRD spectra of (**a**) CNFs, (**b**) CMCNFs, and (**c**) CSNFs.

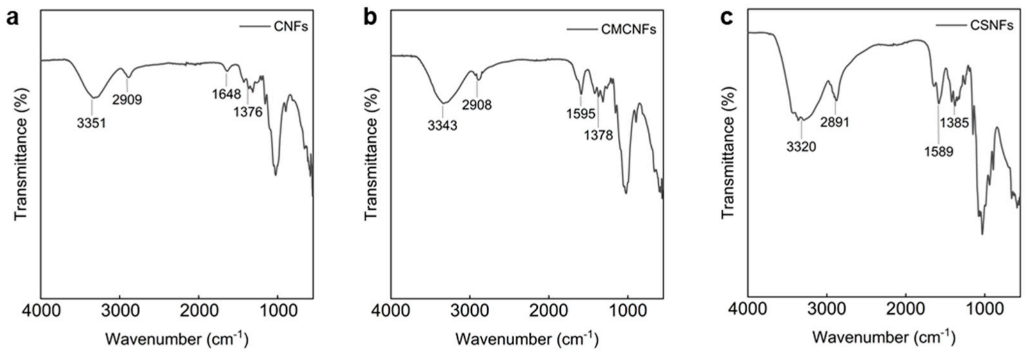

Figure S3. FTIR spectra of (**a**) CNFs, (**b**) CMCNFs, and (**c**) CSNFs.

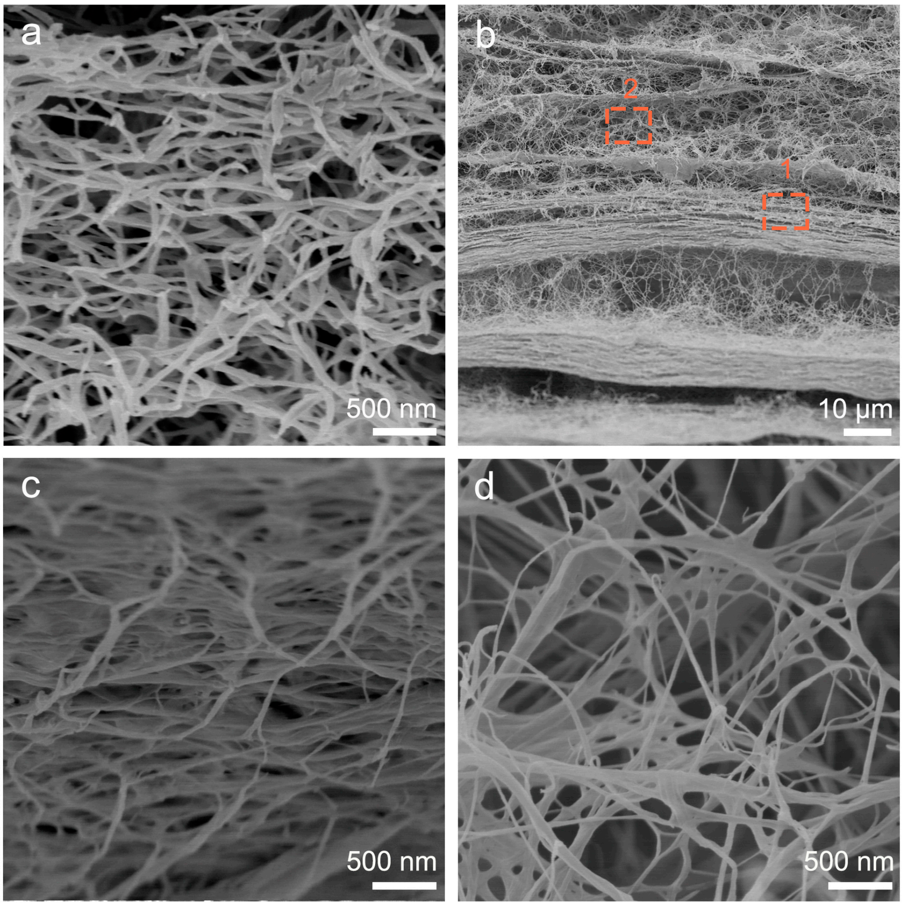

Figure S4. **Synthesis of BC/CHNF hydrogel**: (**a**) SEM image of CHNFs; (**b**) the cross-section images of BC/CHNF hydrogel; (**c**) the magnified cross-section images of box 1 in the Figure S4b; (**d**) the magnified cross-section images of box 2 in the Figure S4b.

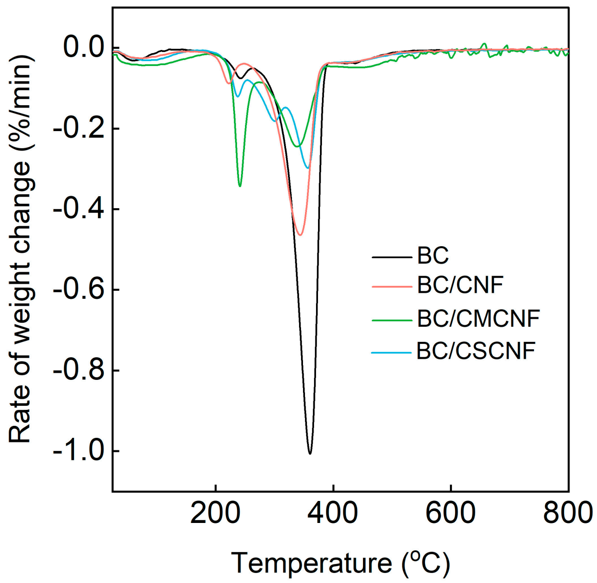

Figure S5. The derivative thermogravimetry curves of BC/BMNF composite hydrogels.

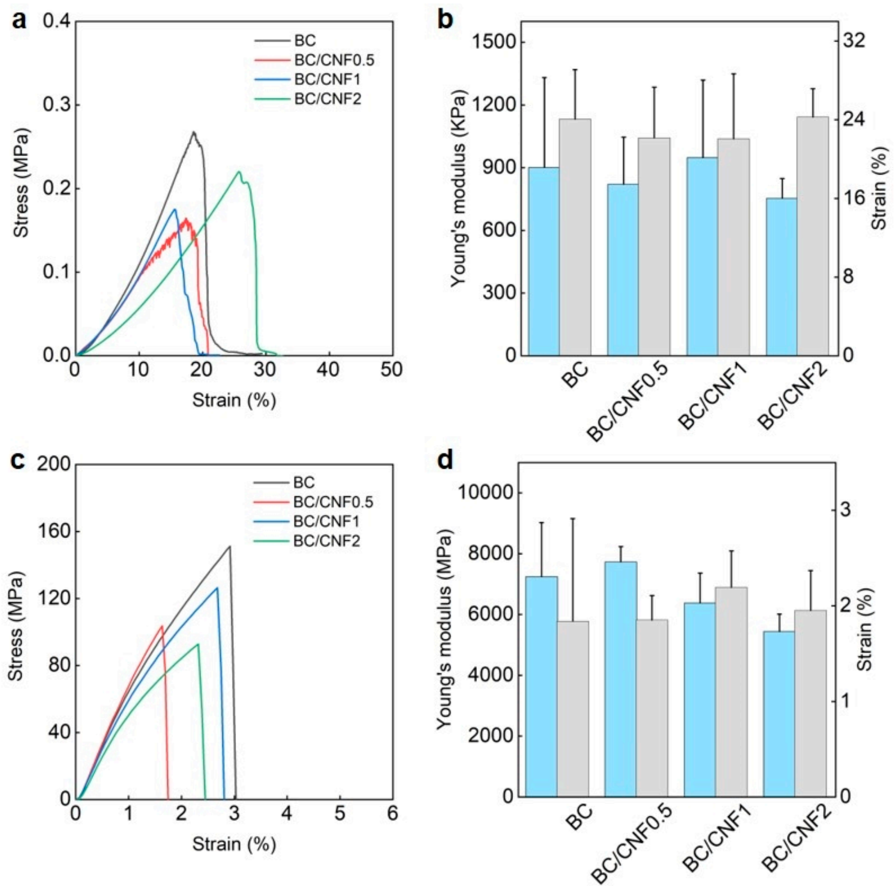

Figure S6. Mechanical properties of BC/CNF hydrogels: (**a**) the tensile stress-strain curves of BC/CNF hydrogels and (**b**) their corresponding Young's moduli and strains; (**c**) the tensile stress-strain curves of BC/CNF films and (**d**) their corresponding Young's moduli and strains; the concentrations of CNFs in the culture media were changed from 0.5 to 1 and 2 wt%, which correspond to the hydrogels of BC/CNF0.5, BC/CNF1.0, and BC/CNF2.0, respectively.

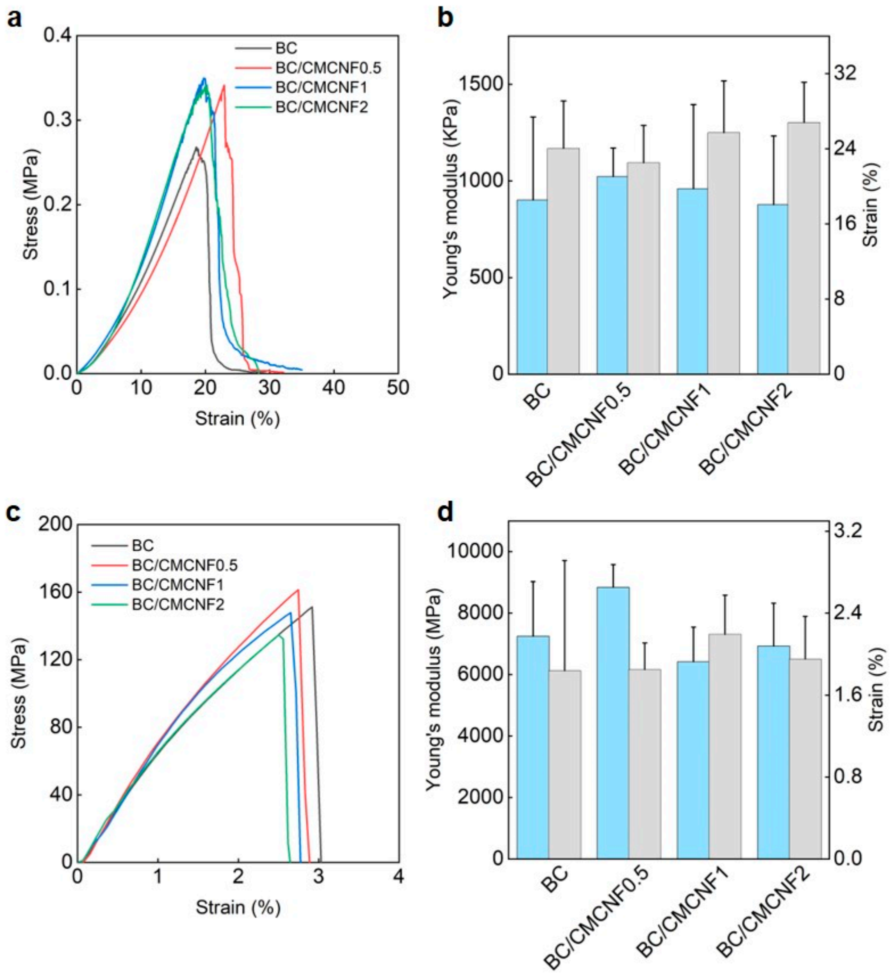

Figure S7. Mechanical properties of BC/CMCNF hydrogels: (**a**) the tensile stress-strain curves of BC/CMCNF hydrogels and (**b**) their corresponding Young's moduli and strains; (**c**) the tensile stress-strain curves of BC/CMCNF films and (**d**) their corresponding Young's moduli and strains; the concentrations of CMCNFs in the culture media were changed from 0.5 to 1 and 2 wt%, which correspond to the hydrogels of BC/CMCNF 0.5, BC/CMCNF1.0, and BC/CMCNF2.0, respectively.

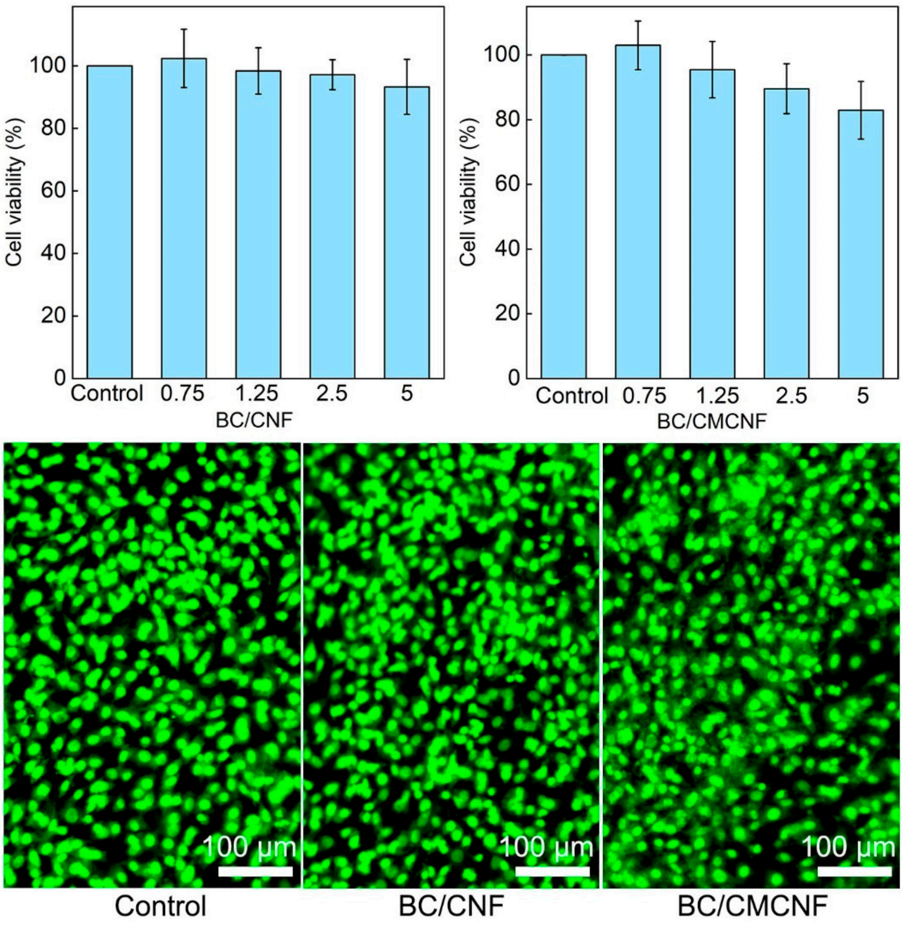

Figure S8. Cytotoxicity of BC/CNF and BC/CMCNF hydrogels evaluated via (**a**) MTT and (**b**) AO/EB staining assays.

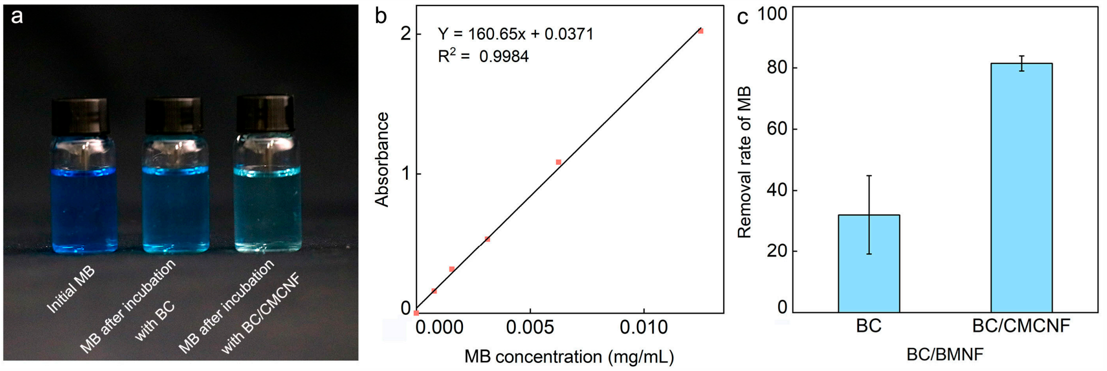

Figure S9. Dye-adsorption capability: (**a**) the photograph of the initial MB solution, and the MB solution after incubation with dried BC and BC/CMCNF films; (**b**) the calibration curve of MB solution; (**c**) the MB adsorption capacity of BC and BC/CMCNF hydrogels.
